# Hysterectomy for the Management of Chronic Endometritis With Keratinising Squamous Metaplasia in an Ovariectomised Pet Sow: A Case Report

**DOI:** 10.1155/crve/9995968

**Published:** 2025-11-19

**Authors:** Eilidh E. Thomson, Julianna R. Gallo, Heather J. Kamataris, Kayla N. Russell, Claire M. Loughran, Giulia Dalla Serra, Andrea Del Rincon, Joseph P. Cassidy, Marijke E. Beltman, Emmet T. Kelly

**Affiliations:** School of Veterinary Medicine, University College Dublin, Belfield, Dublin 4, Ireland

**Keywords:** case report, endometritis, keratinisation, ovariectomy

## Abstract

An 8-year-old female pig was presented to the University College Dublin Veterinary Hospital in February 2025 with chronic intermittent vulval discharge, depression and inappetence. The sow had previously undergone a laparoscopic bilateral ovariectomy in October 2023, with no complications; thus, the development of uterine disease would be highly unusual. Upon clinical examination, there was a moderate amount of mucopurulent vaginal discharge. Transabdominal ultrasonography was suggestive of endometritis secondary to cystic endometrial hyperplasia. A hysterectomy was performed, and histopathological examination of the uterus confirmed cystic endometrial hyperplasia, as well as chronic endometritis with marked keratinising squamous metaplasia of the endometrium and formation of keratinised ‘uteroliths'. There was no evidence of any hormone-producing ovarian tissue in the abdomen. This squamous metaplasia and keratinisation have never been described before in sows, with uterine disease being incredibly rare in an ovariectomised animal with no ovarian remnants present. It may be prudent to consider ovariohysterectomy in sows, especially those with risk factors associated with chronic subclinical endometritis, for example, older animals.

## 1. Case Presentation

A sow, estimated to be 8 years old, presented to the University College Dublin Veterinary Hospital (UCDVH) on 12 February 2025 to investigate chronic intermittent vulval discharge which the owner associated with periods of inappetence and depression. The sow came from a rescue farm and had undergone routine laparoscopic ovariectomy at UCDVH 18 months previously. The owner described regular reproductive cycles prior to ovariectomy and sporadic changes in her demeanour with intermittent vulval discharge postsurgery. She had been administered a course of amoxicillin–clavulanic acid (of unknown dose and route) prior to admittance, with no improvement noted in her condition.

On initial clinical examination, she was quiet, alert and responsive with a normal respiratory rate (24 bpm) and no increased effort. Her heart rate (52 bpm) and temperature (38.2°C) were also normal. She weighed 78 kg, similar to that recorded at the time of ovariectomy surgery in 2023 (72 kg). A moderate amount of mucopurulent vaginal discharge was noted, but no abnormalities were noted on a vaginal examination.

## 2. Diagnosis and Treatment

### 2.1. Diagnostics

A dipstick urinalysis showed a pH of 7 with trace amounts of protein, bilirubin and blood.

A venous blood sample was taken from the auricular vein under sedation and haematology, biochemistry and blood gas were performed. Abnormalities on her biochemistry included a slight increase in CK (636 U/L, reference range: < 50 U/L) and AST (25 U/L, reference range: 10–14 U/L), thought to be caused by recent transportation and/or recent increased recumbency. There was also mild hyperalbuminaemia (45.6 g/L, reference range: 16–38g/L), likely due to mild dehydration, and hypoglobulinaemia (19.6 g/L, reference range: 24–57g/L). Additionally, there was a mild decrease in urea (2.4 mmol/L, reference range: 2.7–8mmol/L) and creatinine (82.47 *μ*mol/L, reference range: 88–178*μ*mol/L), thought to be due to muscle loss or recent protein malnutrition. Her GGT (66 U/L, reference range: < 55 U/L) and glucose (7.98 mmol/L, reference range: 3.6–5.3mmol/L) were insignificantly raised.

Her haematological abnormalities included a leukopaenia (8.39 × 10^9^/L, reference range: 11‐22 × 10^9^/L), mainly lymphopaenia (3.36 × 10^9^/L, reference range: 4.2‐13 × 10^9^/L) and a mildly decreased haematocrit of 29% (reference range: 32%–55%). She also had metabolic alkalosis (pH 7.568, reference range: 7.35–7.45), thought to be caused by the sedation, and a mildly high lactate (2.33 mmol/L, no reference range available although one study found a median of 1.2 mmol/L in sampled pigs [1]), likely due to some degree of tissue damage or mild dehydration.

**Figure 1:**
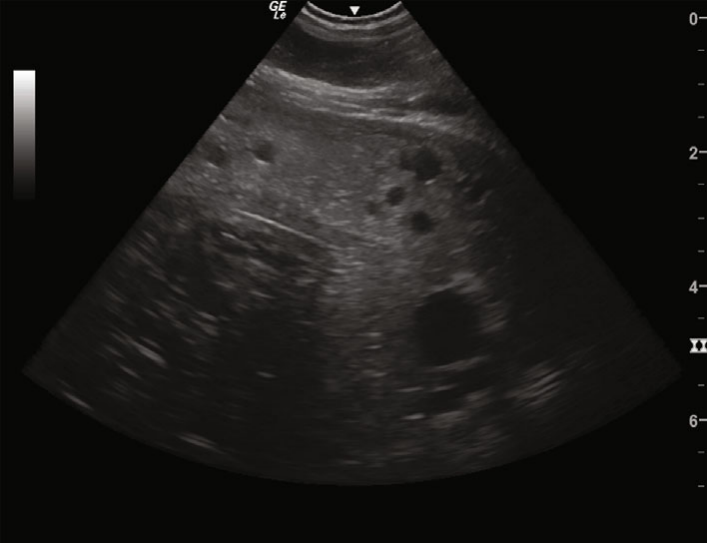
Ultrasound image showing the anechoic intramural lesions.

Transabdominal ultrasonography was performed under sedation, with the sow in lateral recumbency. Her inguinal area and left flank were clipped, and alcohol was used to enhance the image; the uterus was located just cranial to the flank fold. It revealed the uterine parenchyma as heterogeneous with intramural fluid-filled lesions compatible with endometrial cysts and concurrent possible fibrotic/mineralised areas (Figure 1). These changes were consistent with cystic endometrial hyperplasia (CEH). There was also a mild peritoneal effusion adjacent to the uterus, most likely inflammatory in nature.

### 2.2. Anaesthesia

Premedication was achieved with xylazine (2 mg/kg), butorphanol (0.2 mg/kg) and ketamine (5 mg/kg) intramuscularly (im). Once sedated, a 20G 32-mm catheter was placed in the right lateral auricular vein. Anaesthesia was induced with ketamine (2 mg/kg) intravenously (iv) and endotracheal intubation, with a 9-mm tube, was performed after lidocaine was topically applied to the larynx. Anaesthesia was maintained with isoflurane and warmed Hartmann's fluids were administered iv at 5 mL/kg/h throughout anaesthesia.

Analgesia was achieved using paracetamol 10 mg/kg per os preoperatively. Perioperatively, butorphanol 0.1 mg/kg and a ketamine constant rate infusion (CRI) 10 mcg/kg/min were given iv. Xylazine 0.12 mg/kg iv was administered in response to increased systolic arterial pressure, and a xylazine CRI was then started at a rate of 0.2 mcg/kg/min and adjusted as required throughout the surgery. Both CRIs were stopped 30 min before the end of anaesthesia. Hypotension during the anaesthetic was treated with fluid boluses and an adrenaline infusion, 0.05–0.17 mcg/kg/min. Active warming, with a forced air warmer (43°C), was provided throughout anaesthesia and postoperatively. Although hypothermic at the end of the procedure, the body temperature had risen to 37°C, and the sow was bright and active 1 h postextubation.

### 2.3. Surgery

**Figure 2:**
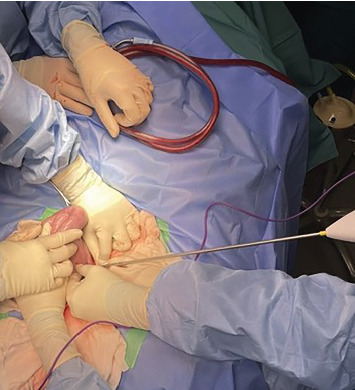
Ligation during surgery.

**Figure 3:**
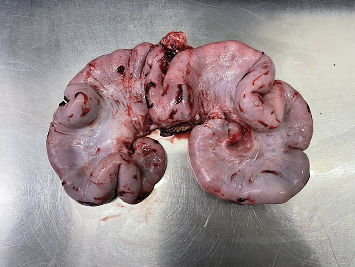
Excised uterus, showing an exposed ‘uterolith'.

The sow was placed in dorsal recumbency, and her caudal abdomen was clipped, aseptically prepared and appropriately draped. A caudal ventral midline approach was performed; a 10-cm incision revealed the uterus in the caudal aspect of the abdomen. Upon inspection, the uterine wall was thickened and featured multiple firm, granular intraluminal material. The mesometrial vessels of the left uterine horn were ligated using a combination of four-metric polydioxanone and a vessel-sealing device (LigaSureTM) (Figure 2). A small remnant which was suspected to be ovarian tissue was identified at the tip of the right uterine horn, and a similar ligation technique was applied to the mesometrial vessels on the right side. Transfixation ligatures were placed on the uterine body immediately cranial to the internal cervical os, followed by sealing with LigaSureTM. To invert the cervical mucosa, a Parker–Kerr suture pattern was applied using five-metric polydioxanone. The uterus was excised (Figure 3), and all ligated pedicles were inspected to ensure adequate haemostasis before closure. The abdomen was closed in three layers: the linea alba with five-metric polydioxanone in a continuous pattern, the subcutaneous tissue with three-metric polydioxanone in a continuous pattern and the skin with three-metric poliglecaprone in an intradermal pattern. An external stent was placed prior to recovery.

### 2.4. Postoperative Care

Immediately postoperatively, meloxicam (0.4 mg/kg) was given iv then im once daily over the four subsequent days. She also received amoxicillin/clavulanic acid (9.7 mg/kg) im once daily for 5 days. The stent was removed 4 days postoperatively, and she was discharged the following day with an excellent prognosis. All medications used, including withdrawal times, were noted in the UCDVH medicine book and also in a prescription sent home with the owner, as per Veterinary Medicinal Products Regulations in Ireland.

## 3. Outcome and Follow-Up

The sow remained clinically normal postoperatively. Histopathological examination was carried out: The suspected ovarian remnant tissue revealed distorted transverse sections of dilated oviducts within a well-vascularised fibromuscular stroma. No ovarian tissue was found.

**Figure 4:**
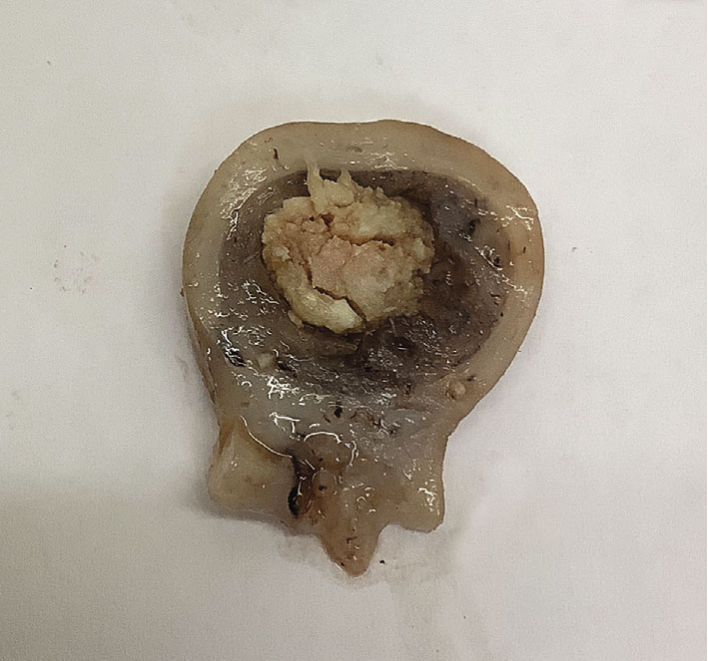
Cut section of uterus, showing thickened endometrium and a keratinised ‘uteroliths.

**Figure 5:**
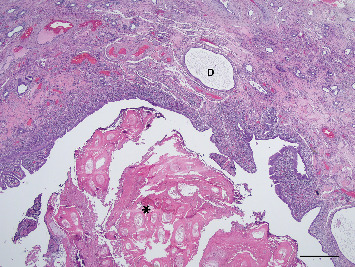
Photomicrograph illustrating dense, laminated, keratinous material (⁣^∗^) within the lumen of the chronically inflamed uterus. Dilated gland (D) evident in endometrium. Haematoxylin and eosin (H&E) stain, scale bar = 500* μ*m.

**Figure 6:**
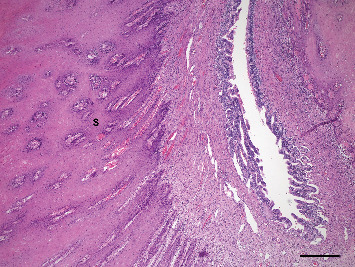
Photomicrograph illustrating thick squamous metaplasia (S) of uterine gland epithelium. Arrows indicate adjacent uterine epithelial surface. H&E stain, scale bar = 250* μ*m.

**Figure 7:**
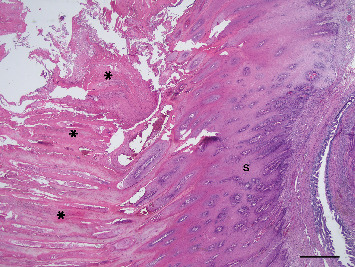
Photomicrograph illustrating florid keratinisation (⁣^∗^) of the squamous metaplastic surface (S) of a uterine gland lumen. H&E stain, scale bar = 500* μ*m.

Examination of the uterus (Figure 4) revealed irregularly and markedly thickened endometrium with dilated glands that were variously optically empty or contained fibrinocellular exudate and mucous. Dense infiltration of admixed lymphocytes and plasmacytes, along with oedema and fibrosis, was noted in the surrounding stroma. Marked keratinising squamous metaplasia of the epithelium was observed in many glands. Thick irregularly hyperplastic layers of stratified squamous epithelium undergoing surface hyperkeratosis had resulted in florid laminations of keratin bulging into the uterine lumen (Figures 5, 6, and 7). Within this keratinised material, fibrinocellular exudate, dystrophic mineralisation and occasional clusters of basophilic bacteria were noted.

The histopathological diagnosis was marked by chronic endometritis featuring CEH and squamous metaplasia with keratinisation.

On follow-up, 2 months after the procedure, the owner reported no clinical signs of depression, inappetence or vulval discharge and no postoperative complications.

## 4. Discussion

Endometritis is characterised by inflammation of the endometrium [2] and is usually seen postpartum in pigs [3]. Subclinical forms are not uncommon in the pig and can lead to the development of a pyometra [4]. The most common uterine lesion to affect pigs (75%) in one study is CEH, with pyometra seen in 9.3% of the same population [5]. Older pigs are more likely to develop uterine lesions [6], with nulliparous sows more prone to developing CEH [7]. To the authors' knowledge, keratinising squamous metaplasia with the development of ‘uteroliths' has not been described in domestic species.

Metaplasia is the change from one differentiated cell type into another of the same germ line, and typically, squamous metaplasia is a reparative response to chronic inflammation–injury (e.g., smoking in humans [8]), hormone imbalance (e.g., oestrogen-induced in the prostate gland [9]) or trauma [10]. Keratinocyte growth factor is expressed by porcine endometrial epithelia [11], and our working hypothesis is that, in the absence of detectable hormonal stimulation, longstanding subclinical endometritis present likely prior to ovariectomy resulted in widespread squamous metaplasia of the uterine glandular epithelium. Florid keratinisation of this altered glandular surface formed the keratinised ‘uteroliths' that bulged into the lumen. While there are reports of ‘uterine stones' in human patients, these were calculi of calcium carbonate or phosphate [12] and not keratinised, and similar ‘inspissated uterine secretions' are described in cattle [13].

The question remains as to whether a full ovariohysterectomy should be performed in pet/rescue pigs, that are more likely to be older and/or nulliparous and thus more susceptible to developing [5, 6] or may have existing uterine pathology. Due to the fact that laparoscopic ovariectomy is associated with reduced surgical trauma and reduced recovery time [14], this less invasive surgery may still be the preferred option in elective cases. Another possibility would be to perform ultrasonography to detect uterine lesions prior to routine ovariectomy and thus make an informed decision to switch to ovariohysterectomy in select cases.

This case report has potential implications in assessing uterine pathology in other species. While CEH and pyometra are amongst the most common uterine pathologies in the bitch [15], they are rarely reported following ovariectomy [16]. Squamous metaplasia is reported in intact bitches with the CEH–pyometra complex [17] but without keratinisation. This report expands our knowledge on the pathological processes that may occur in the chronically inflamed uterus, lesions that may be reported in other species in the future.

Limitations of this case report include its singular and retrospective nature. In the future, studies could evaluate any differences in long-term outcomes associated with either elective ovariectomy or ovariohysterectomy in pigs to determine the best approach in these cases as pet pig ownership becomes more popular. Bacteriology of the uterine contents would have been beneficial to perform and thus advise on the treatment of future cases; however, unfortunately, in this sow, antibiotics had been administered prior to admission. Further research needs to be done on the pathological process of squamous metaplasia in the endometrium and the formation of keratinised ‘uteroliths' in different domestic animal species.

## 5. Conclusions

Endometrial squamous metaplasia with ‘uterolith' formation in domestic animals has not previously been described in the literature. Furthermore, this case was also unusual due to the development of endometritis postovariectomy indicating that subclinical endometritis should be considered when performing routine ovariectomies in pigs. The findings may also be transferable to other species, such as dogs.

## Data Availability

The data that supports the findings of this study are available from the corresponding author upon reasonable request.
